# A Fast Attention-Guided Hierarchical Decoding Network for Real-Time Semantic Segmentation

**DOI:** 10.3390/s24010095

**Published:** 2023-12-24

**Authors:** Xuegang Hu, Jing Feng

**Affiliations:** 1 School of Communications and Information Engineering, Chongqing University of Posts and Telecommunications, Chongqing 400065, China; huxg@cqupt.edu.cn; 2 Chongqing Key Laboratory of Signal and Information Processing, Chongqing University of Posts and Telecommunications, Chongqing 400065, China

**Keywords:** real-time semantic segmentation, attention mechanism, encoder–decoder network, feature fusion

## Abstract

Semantic segmentation provides accurate scene understanding and decision support for many applications. However, many models strive for high accuracy by adopting complex structures, decreasing the inference speed, and making it challenging to meet real-time requirements. Therefore, a fast attention-guided hierarchical decoding network for real-time semantic segmentation (FAHDNet), which is an asymmetric U-shaped structure, is proposed to address this issue. In the encoder, we design a multi-scale bottleneck residual unit (MBRU), which combines the attention mechanism and decomposition convolution to design a parallel structure for aggregating multi-scale information, making the network perform better at processing information at different scales. In addition, we propose a spatial information compensation (SIC) module that effectively uses the original input to make up for the spatial texture information lost during downsampling. In the decoder, the global attention (GA) module is used to process the feature map of the encoder, enhance the feature interaction in the channel and spatial dimensions, and enhance the ability to mine feature information. At the same time, the lightweight hierarchical decoder integrates multi-scale features to better adapt to different scale targets and accurately segment objects of different sizes. Through experiments, FAHDNet performs outstandingly on two public datasets, Cityscapes and Camvid. Specifically, the network achieves 70.6% mean intersection over union (mIoU) at 135 frames per second (FPS) on Cityscapes and 67.2% mIoU at 335 FPS on Camvid. Compared to the existing networks, our model maintains accuracy while achieving faster inference speeds, thus enhancing its practical usability.

## 1. Introduction

Real-time semantic segmentation [1] is a challenging task in computer vision [2] that aims to classify each pixel in an image and assign it to its corresponding semantic class. In practical applications, real-time semantic segmentation algorithms are often combined with sensor networks. Sensor networks provide real-time environment-aware data, such as images, videos, or other sensor information. The real-time semantic segmentation algorithm uses these data for scene analysis and object recognition to realize the real-time understanding and intelligent analysis of the environment. For example, intelligent transportation systems use sensor networks to sense traffic flow, road status, and other information. The real-time semantic segmentation algorithm can analyze the image data collected by sensor networks and perform semantic segmentation on roads, vehicles, pedestrians, etc., to realize the functions of traffic flow statistics, vehicle detection and recognition, and pedestrian behavior analysis. This combination has essential application value in autonomous driving [3], robot perception [4,5], intelligent monitoring, and so on.

In recent years, the rapid development of microelectronics technology has improved the efficiency of semantic segmentation tasks. However, with the increasing demand, the requirements for semantic segmentation tasks have become more and more stringent. Some complex real-time semantic segmentation models [6,7] have excellent performance in segmentation accuracy. Still, it has problems such as a large amount of computation and slow inference speed, which cannot meet the requirements of low-performance IoT devices for real-time tasks, especially for applications such as autonomous vehicles and mobile devices, which have limited computational resources and require efficient interaction, strict inference speed is usually needed to achieve fast interaction. Although the improvement of computing power can alleviate some problems, there are still many challenges. Therefore, we need further research and innovation to explore more effective models and algorithms to meet these needs. Designing efficient real-time semantic segmentation methods is still an important research direction.

**Figure 1:**
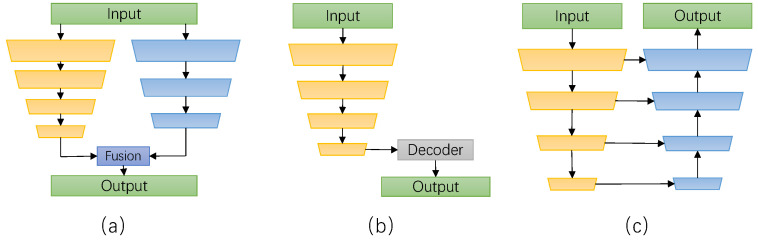
(**a**) Depicts a double-branch structure; (**b**) illustrates an asymmetric encoder–decoder structure; (**c**) showcases a U-shaped structure. The yellow part here is the encoding part, and the blue part is the decoding part.

To solve the aforementioned problem, researchers have taken a series of measures to improve the inference speed. They mainly include the following two directions. The first direction is the design of lightweight network structure. Researchers have proposed three network structures based on convolutional neural networks [8] for real-time semantic segmentation. The first type is the dual branch structure [9,10,11], as shown in Figure 1a, which has become the main structure for real-time semantic segmentation. This structure improves semantic segmentation performance significantly. However, it is necessary to attach great importance to the fusion of the two branch features, as inappropriate fusion may reduce performance. The second type is the lightweight asymmetric encoder–decoder structure [12,13,14,15], as shown in Figure 1b. This structure achieves fewer parameters and computational complexity, which makes it suitable for real-time semantic segmentation tasks. Still, It may result in relatively low accuracy of segmentation results. The third type is the U-shaped structure [16,17,18,19,20], as shown in Figure 1c. It enhances global context understanding and improves the grasp of semantic information in images by using deep features of encoders and shallow features of skip connections. This structure performs excellently in terms of segmentation accuracy. Nonetheless, the U-shaped structure model still has the problems of high computational complexity and high memory consumption. The second direction is network pruning and compression [21]. Through network pruning and compression technology, the redundant model parameters and computation are removed to reduce the size and complexity of the model, so as to improve the inference speed. This often leads to the reduction of the parameters and the complexity of the model, which may lead to the loss of segmentation accuracy. Although real-time is very important, it cannot be at the expense of the accuracy of semantic information. Accuracy is still the most important index by which to evaluate the quality of the model. The reason is that the higher the segmentation accuracy of the model, the stronger the scene analysis ability and the more practical the use value. For example, good scene perception ability helps robots provide accurate visual information and helps robots understand and process the environment. However, if the inference speed of the model far exceeds the actual demand, it will waste computing resources and may increase the complexity and cost of the system. Therefore, the real-time semantic segmentation algorithm also needs to achieve an excellent trade-off between the inference speed, accuracy, and model parameters.

**Figure 2:**
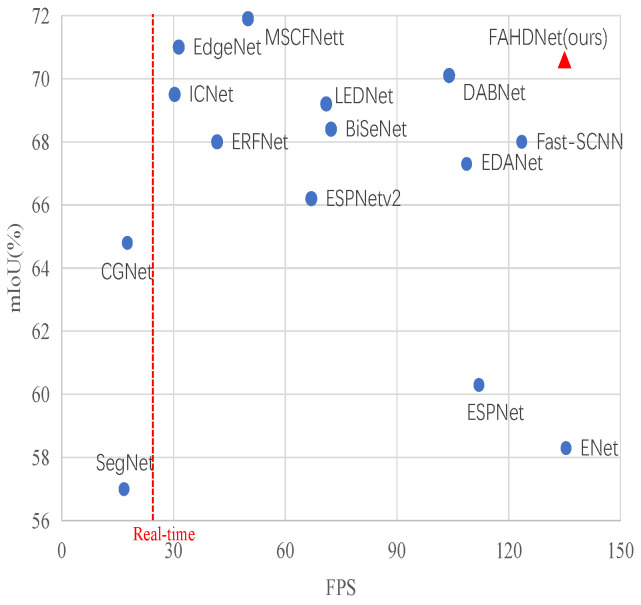
Model inference speed and segmentation accuracy on the Cityscapes dataset. In the diagram, the red triangle represents our method, the blue dots represent other methods, and the red dashed line represents the minimum requirements for real-time semantic segmentation.

Based on the above analysis, our research focuses on solving the balance problem mentioned above. The main idea is to improve the inference speed and reduce the memory occupation without reducing the segmentation accuracy significantly. To solve this problem, we have improved the U-shaped structure mentioned above. In order to solve the problems of high computing and memory consumption of the U-shaped structure, our model uses the asymmetric U-shaped structure and designs a lighter and more efficient hierarchical decoder, taking into account the feature extraction ability and resource occupation. Secondly, this paper also improves the feature extraction module. Different from the existing feature extraction module, our module combines the residual module and deconvolution to ensure the lightweight of the module and does not cause a significant decline in accuracy. In addition, in order to improve the robustness and generalization ability of the model, we also introduce auxiliary losses in the training phase. On account of the above improvements, we propose a novel model that achieves a good balance of inference speed, accuracy, and model parameters. Figure 2 shows the comparison of our model with other models on the Cityscape dataset.

**Figure 3:**
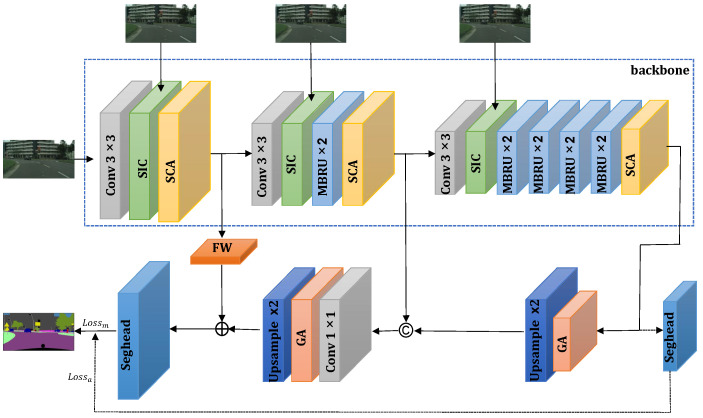
The procedure of our proposed FAHDNet. ‘C’ denotes the concatenation, ‘+’ denotes the pixel level addition operation. The blue box represents the downsampling backbone network of the network, while the black dashed arrow indicates that it only exists during training and is discarded during the inference process.

A fast attention-guided hierarchical decoding network for real-time semantic segmentation (FAHDNet) is proposed in this paper. The overall structure is shown in Figure 3. In the encoder, we use multi-scale bottleneck residual units (MBRU) to extract the fundamental features of the image. To enhance the segmentation performance of the model for spatially unevenly distributed objects, we introduce the spatial information compensation (SIC) module, which successfully integrates rich spatial details from the original image with downsampled features to compensate for the loss of minority class information. In the decoder, a global attention (GA) module and feature weighting (FW) module are employed to guide feature upsampling, enabling sufficient global information interaction and obtaining more accurate segmentation results. In summary, the main contributions of this paper are as follows:

- We design a lightweight real-time semantic segmentation network, which can achieve competitive segmentation accuracy with lower computational overhead and fewer network parameters, making the segmentation model more suitable for resource-constrained edge devices.
- A multi-scale bottleneck residual unit (MBRU) is proposed. MBRU makes full use of factorized convolution to reduce computational overhead and increase the inference speed. Moreover, it constructs a parallel structure to extract semantic information at different scales effectively to obtain multi-scale features. Then, the channel attention weight of multi-scale feature maps is calculated for feature re-calibration.
- An efficient SIC module allows the network to utilize the original input and combine the attention module to compensate for the lost spatial texture information in the downsampling process, achieving the maximum improvement effect with the minor parameters.
- A lightweight hierarchical decoder is designed, reducing the semantic gaps in multiple resolution feature maps in feature reconstruction. The decoder also combines the GA and FW modules that adaptively enhance multi-level features to balance the feature maps in feature fusion, so as to solve the local fuzzy problem in segmentation.

## 2. Related Work

### 2.1. Attention Mechanism

Attention mechanism is a data processing method widely used in machine learning tasks, including natural language processing, image processing, and speech recognition. According to the application method and location of attention weights, attention mechanisms can be divided into three types: channel domain, spatial domain, and hybrid domain.

The channel domain attention mechanism is a method of channel selection. In convolutional neural networks, the convolutional operation usually focuses on the receptive field, and the information of all channels is fused by default. Therefore, SENet [22] introduced the SE module, which focuses attention between channels, aiming to let the model learn the weight distribution between different channels. FcaNet [23] improves the squeeze part of SE module and proposes an efficient multispectral channel attention framework. ECANet [24] improves the excitation in SENet to avoid the negative effects of dimensionality reduction on channel attention while enhancing the efficiency of the dependency calculation. The spatial domain attention mechanism is an adaptive spatial region selection mechanism. For example, the EMA module proposed by EMANet [25] abandons the process of computing attention maps across the entire image. Instead, it employs the Expectation-Maximization algorithm to learn attention features, thus successfully addressing the issue of high computational complexity. The hybrid domain combines channel attention and spatial attention. For instance, CBAM [26] employs the same attention module to generate channel weights and spatial weights, and then performs weighted processing on the feature maps to generate the final feature map. On the other hand, JAPNet [27] combines spatial attention and channel attention in parallel to effectively extract feature information.

Although the above attention methods are excellent at improving model performance, they are usually accompanied by higher computational costs, resulting in slower reasoning.

### 2.2. Optimize Convolution

Convolution aims to extract useful features from the input image, which is very important for image processing. Therefore, the innovation of convolution is also a research hotspot. These convolutions include asymmetric convolution, dilated convolution, depthwise separable convolution, and the combination of these convolutions, for example, asymmetric dilated convolution [20,28] and depthwise dilated convolution [29,30]. Depthwise separable convolution and asymmetric convolution are called convolution decomposition, which can reduce the parameters and complexity of the model. Using these convolutions is a practical choice for designing a lightweight real-time semantic segmentation model. Compared with the standard convolution, the dilated convolution can increase the receptive field under the convolution kernel with the same size to extract the global information. However, improper use of dilative convolution may also bring negative effects: the loss of local information due to the grid effect or the introduction of information from unrelated regions, eventually leading to unsatisfactory segmentation results. Therefore, when designing the model, we carefully choose the convolution combination suitable for our model.

### 2.3. Feature Fusion

To enhance segmentation performance, many real-time semantic segmentation methods often employ multiple downsampling operations to acquire more contextual information. However, this may lead to the loss of spatial information. To retain more spatial details, some methods attempt to aggregate feature maps from different levels to fuse both contextual information and spatial details. Some of these methods utilize simple operations such as element-wise addition or channel concatenation for feature aggregation. Nevertheless, due to misalignment issues between feature maps at different levels, this may result in a decrease in segmentation performance for smaller objects.

Therefore, SFNet [31] proposes FAM to align and aggregate feature maps of different levels. MSFFM [32] calculates the correlations between pixels in other feature maps by matrix multiplication and uses the correlations as weight vectors for higher-level feature maps. In PIDNet [33], the boundary attention-guided module utilizes boundary features to guide the fusion of details and contextual representations, thereby achieving better semantic segmentation results. The SFA module of SFANet [34] effectively aligns feature maps from adjacent levels and enhances spatial and contextual information during the feature aggregation process. MS-CAM [35] proposes a multi-scale channel attention module to integrate semantic and scale-inconsistent features better. Our model also integrates and fuses features from different levels, enabling the model to better capture key structures and semantic information in the image.

### 2.4. Real-Time Semantic Segmentation

Recently, with the continuous development of new applications, the study of real-time semantic segmentation has become more and more significant. In ICNet, a cascade network is proposed to efficiently use low-resolution semantic information and high-resolution detail information for real-time semantic segmentation. The structure of ShelfNet [36] is similar to that of multiple columns of bookshelves. This design significantly increases the number of pathways from input to output, helping to improve the flow of information within the network. From the point of view of feature redundancy, Ghostnet [37] proposes an innovative Ghost module to generate more feature maps through cheap operation. DFANet [38] is designed as three sub-networks, each of which is used to optimize the output of the previous network, maximizing the interaction and aggregation of features at different levels to improve accuracy in situations with limited computational resources. DWRSeg [39] focuses on expanding the receptive field of feature extraction to realize an ultra-real-time semantic segmentation network. DDRNet proposes a novel bilateral network architecture that combines feature aggregation and a pyramid structure to obtain rich contextual information.

## 3. Proposed Method

In this section, we first present the MBRU and SIC module for joint feature extraction in the encoder, then introduce the GA module for multi-scale feature adaptive fusion in the decoder, and finally, overview the architecture of the proposed FAHDNet.

### 3.1. MBRU Module

The role of the encoder is to convert the input image into a feature representation that subsequent decoders or segmentation heads can use for pixel-level classification. We have designed the MBRU module as a crucial component of the encoder. It can extract rich semantic features, which are essential for identifying different objects and entities in the image, and can significantly reduce confusion and errors in segmentation.

**Figure 4:**
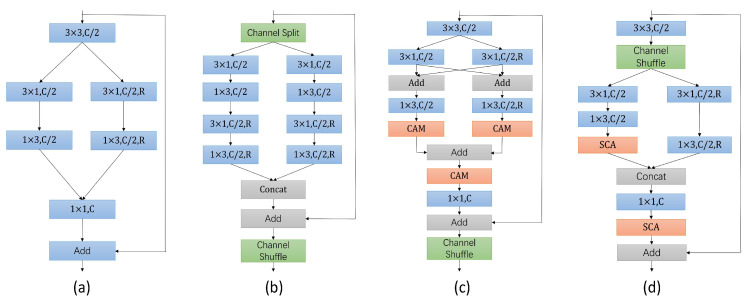
(**a**) Represents the DAB module in DABNet, (**b**) depicts the SS-nbt module in LEDNet, (**c**) illustrates the EAR module in MSCFNet, and (**d**) showcases our MBRU module. ‘C’ represents the number of channels, ‘R’ represents the dilated convolution, and the dilated rate is R.

In Figure 4, we show several classic units for extracting basic features. The DAB module (Figure 4a) has excellent multi-scale feature extraction capability. The SS-nbt module (Figure 4b) uses spatial separation convolution, which reduces computational complexity and makes the model more efficient. In addition, the EAR module (Figure 4c) introduces the CAM to improve the performance and generalization of the model. Based on the advantages of the above modules, we propose the MBRU module (Figure 4d).

Firstly, the MBRU module uses group convolution to reduce the number of channels by half, significantly reducing the parameter count and improving computational efficiency. At the same time, group convolution independently operates on each group, allowing the model to focus more on the local regions of input data, learn more stable and reliable feature representations, and enhance the model’s robustness and feature learning capability. Unlike other models, we choose to perform channel confounding in the front part of the module, taking channel correlations into account earlier, thereby influencing feature learning within the module to obtain richer feature representations. Later, the features are processed by two separate branches. The left branch first utilizes 3 × 3 decomposed convolutions, employing small convolutional kernels to encourage the network to learn richer features, thereby enhancing generalization capabilities. A simple channel attention (SCA) module is used to adjust the information flow between branches, increasing flexibility. The right branch employs dilated 3 × 3 decomposed convolutions to handle information at different scales. Cascade fusion of left and right branch features comprehensively captures all input data aspects and patterns and improves network expression ability. Considering the difference between left and right branch information attributes, point convolution and SCA modules are used to integrate semantic and contextual information better. The final residual connection preserves the original feature information and helps mitigate the degradation problem. The process of MBRU can be expressed as:

(1) $$X=C^{CS}\left(C^{3\times 3,G}\left(X_{in}\right)\right)$$

(2) $$X_{l}=C^{SCA}\left(C^{1\times 3}\left(C^{3\times 1}\left(\left(X\right)\right)\right)\right)$$

(3) $$X_{r}=C^{1\times 3,R}\left(C^{3\times 1,R}\left(\left(X\right)\right)\right)$$

(4) $$X=C^{SCA}\left(C^{1\times 1}\left(C^{CAT}\left(\left(X_{l},X_{r}\right)\right)\right)\right)$$

(5) $$X_{out}=X+X_{in}.$$

Here, $C^{3\times 3}\left(\cdot \right)$ refers to the 3×3 convolution, $C^{CS}\left(\cdot \right)$ refers to channel shuffle, $C^{3\times 1}\left(\cdot \right)$ and $C^{1\times 3}\left(\cdot \right)$ refer to the asymmetric decomposition convolution, respectively, G denotes group convolution, R is dilated convolution, $C^{SCA}\left(\cdot \right)$ represents the channel attention module, and $C^{CAT}\left(\cdot \right)$ indicates a concatenate connection.

**Figure 5:**
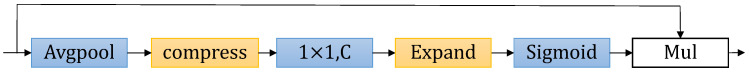
SCA module. ‘Avgpool’ represents average pooled downsampling, ‘Sigmiod’ represents activation function. ‘Compress’ refers to compressing features, while ‘extend’ means extending features.

Among them, the SCA module, as shown in Figure 5, realizes the interaction of global information through the compression and conversion of features. It is multiplied with the original input feature to weigh the output of the tuned model better to capture the relevance and importance of the input data.

### 3.2. SIC Module

The urban road scene involves the relative position relationship of road structure, vehicles, and pedestrians, which is a complex environment. However, the downsampling process in the encoder can lead to the loss of spatial information, making it more challenging to extract target relative positional relationships, especially in pixel-level semantic segmentation tasks, which may have an adverse impact on accuracy.

To better obtain rich spatial information, BiSeNet uses auxiliary branches to capture more local details, but this also increases the complexity of the network, resulting in higher training costs. DANet employs a self-attention mechanism to capture spatial and channel dependencies between features. Still, unfortunately, self-attention mechanisms often introduce a lot of parameter calculations and are not suitable for real-time inference.

**Figure 6:**
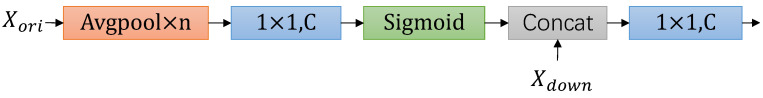
SIC module. Here, $X_{ori}$,$X_{down}$ represent the original input feature and the subsample feature.

Considering the above problems, we propose the SIC module, as shown in Figure 6, which is used after the convolution unit. It combines raw images and spatial attention mechanisms. Firstly, the adaptive average pooling operation is carried out on the original input image to reduce the spatial dimension of the feature map and extract the key feature information at the same time. Then, the spatial attention mechanism is used to dynamically allocate each pixel’s attention weight to improve the segmentation accuracy. We carry out cascading fusion to enhance the interaction with the down-sampled features in the backbone network. Finally, point convolution is used to reduce the difference between the two sets of feature attributes, so as to achieve better semantic segmentation. The module structure can be expressed as:

(6) $$X_{ori}=\sigma \left(C^{1\times 1}\left(C^{pool\times n}\left(X_{ori}\right)\right)\right)$$

(7) $$X_{out}=C^{1\times 1}\left(C^{cat}\left(X_{ori},X_{down}\right)\right).$$

Here, $X_{ori}$,$X_{down}$ represent the original input feature and the subsample feature, $C^{pool\times n}\left(\cdot \right)$ indicates that the average pooling undersampling is performed for n times, and n indicates the n stage of the encoder, σ(·) represents the sigmoid activation function, $C^{1\times 1}\left(\cdot \right)$ represents the 1 × 1 standard convolution.

### 3.3. GA Module

**Figure 7:**
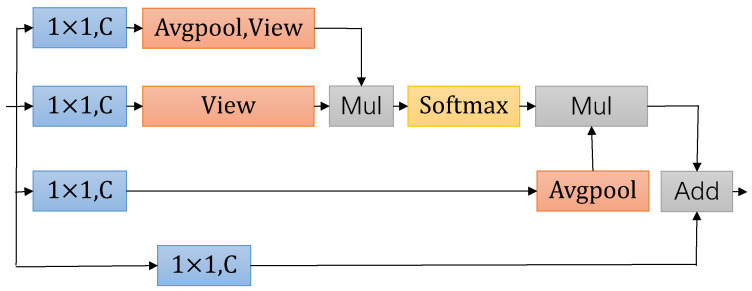
GA module. ‘Avgpool’ represents average pooled downsampling, ‘Softmax’ represents softmax activation function. ‘View’ represents the transformation of features in terms of dimensionality.

Our network only downsamples the input resolution to one-eighth. While this helps in making the model lightweight and more efficient in terms of computation during training and inference, it leads to issues of misclassification in the segmentation results due to the limited receptive field and the limited understanding of semantic information. To capture richer semantic information, it is often necessary to consider how to expand the receptive field, enabling the recognition of semantic relationships between distant pixels for accurate pixel classification into their semantic categories. The DeepLab [40] series of works mainly focus on dilation convolution, such as the ASPP module proposed in DeepLab v2. CCNet [41] employs a unique cross-attention module, which collects contextual information for all pixels along the pixel-crossing paths. Therefore, we propose the GA module to enhance the network’s ability to understand the global scenario, as shown in Figure 7.

Our module is divided into several branches. Each branch is nonlinearly transformed at the beginning by a point convolution with a nonlinear activation function (ReLU). This helps the model capture complex features, allowing it to better distinguish between different semantic categories. Next, the features from the first branch undergo adaptive average pooling and feature compression transformations. They are then subjected to pixel-wise multiplication with the features from the second branch, allowing the aggregation of global information from different dimensions to generate refined feature representations. This set of features associates each pixel with multiple categories using the softmax function, resulting in weight vectors. Subsequently, these weight vectors are multiplied again with the features from the third branch, which have undergone average pooling, to obtain output features. This process integrates both global information and weight vectors, extracting reliable information from the feature map. Finally, the output feature map is combined with the features from the fourth branch, further optimizing the performance of the GA module. It can be expressed as:

(8) $$X_{1}=T\left(C^{pool}\left(C^{1\times 1}\left(X_{in}\right)\right)\right)$$

(9) $$X_{2}=T\left(C^{1\times 1}\left(X_{in}\right)\right)$$

(10) $$X_{2}=\beta \left(C^{Mul}\left(X_{1},X_{2}\right)\right)$$

(11) $$X_{3}=C^{pool}\left(C^{1\times 1}\left(X_{in}\right)\right)$$

(12) $$X_{out}=C^{1\times 1}\left(X_{in}\right)+C^{Mul}\left(X_{2},X_{3}\right).$$

Here, $C^{1\times 1}\left(\cdot \right)$ represents the 1×1 standard convolution. β represents the softmax activation function, T(·) represents the feature compression transpose operation. $C^{pool}\left(\cdot \right)$ indicates adaptive average pooling. $C^{Mul}\left(\cdot \right)$ is the product of the pixel level multiplication of features.

### 3.4. Network Architecture

In this section, we will introduce FAHDNet, as proposed in this paper, in detail. Its specific architecture is depicted in Figure 3, and the structural details are provided in Table 1.

The encoder of the network consists of convolutional units, SIC modules, MBRUs, and SCA modules. The encoder is divided into three stages based on the feature resolution size to achieve stage-specific encoding goals. In the first stage, the convolutional unit, SIC module, and SCA module are mainly responsible for completing the first stage of encoding tasks. This stage mainly involves preliminary feature extraction and processing of the input data. In the second stage, the convolutional unit, SIC module, MBRUs, and SCA module jointly complete specific encoding tasks. This stage mainly carries out further feature extraction and coding for the data processed in the first stage. In the third stage, the convolutional unit, SIC module, MBRUs, and SCA module also work together to continue to complete specific encoding tasks. This stage mainly involves advanced feature extraction and the encoding of the data processed in the first two stages. Among them, the main purpose of the convolution units is to reduce the resolution of the input feature image and reduce the redundant visual information through the image downsampling. In order to more accurately extract the relative spatial relationships of the targets and promote information flow between feature channels, we utilized the SIC module after the convolutional units in each stage. This module combines the convolutional results with input features downsampled at different rates to further integrate spatial information. Specifically, the original images are downsampled at rates of 1, 2, and 3. Considering that convolutions with larger receptive fields can capture more information, we use MBRU modules to extract multi-scale features and reuse redundant information after the second and third stages of the SIC module. In these two stages, we employ two and eight MBRU modules, respectively. However, convolutions with a larger receptive field also come with higher computational costs. Therefore, to strike a balance between the number of parameters and accuracy, we adjust by reducing the number of channels. Through experiments, we found that setting the channel numbers for the three stages to 32, 64, and 128, respectively, can achieve this balance. At the end of each stage, the SCA module is employed to enhance the correlation of information between contexts, resulting in more accurate segmentation results.

**Figure 8:**
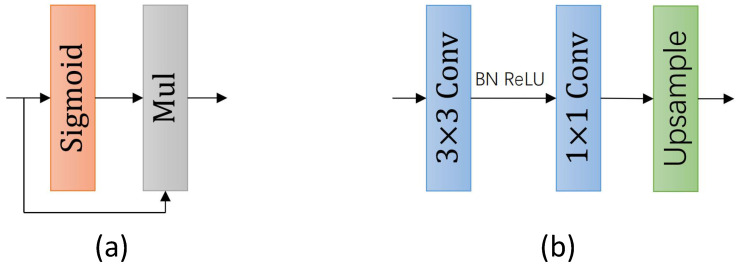
(**a**) The FW module and (**b**) the segmentation head.

The decoder mainly consists of the following modules: the GA module, convolutional units, the FW module, and the segmentation head. The GA module is used to extract global information for better segmentation of complex scenes. The convolutional units aim to reduce feature discrepancies after upsampling and further merge the cascaded features. The FW module (shown in Figure 8a) first processes the input through the Sigmoid function, then multiplies it to achieve non-linear weighting, and finally aggregates it with the features in the decoding stage to extract essential information. The segmentation head (shown in Figure 8b) is composed of standard 3 × 3 convolutions, 1 × 1 convolutions, and bilinear interpolation upsampling operations, and it is located at the end of the decoder. The main function of this module is to restore the feature image to the original image’s resolution, generating the final segmented image. Additionally, we also insert a segmentation head at the end of the third stage as an auxiliary segmentation head. It should be noted that the auxiliary segmentation head is the same as the segmentation head, upsampling after two convolutions. The difference is that the auxiliary segmentation head performs upsampling eight times, and the segmentation head performs twice. The auxiliary segmentation branches are connected by black dotted lines in Figure 3. This segmentation head at this position will be discarded during the inference stage, hence it will not affect the network’s parameter count and segmentation efficiency.

## 4. Experiments

### 4.1. Datasets

The Cityscapes dataset is a large database focused on a semantic understanding of urban street scenes. It consists of approximately 5000 finely annotated images and 20,000 coarsely annotated images. The data were collected from 50 cities over several months, with all images captured during daylight and in good weather conditions. Out of the 5000 finely annotated images, 2975 are used for training, 500 for validation, and the remaining 1525 for testing.

The Camvid dataset is a road scene dataset captured by a camera with a resolution of 960 × 720. It comprises video sequences filmed from the perspective of vehicles, covering both urban and highway environments, with a total of 701 frames. The dataset includes 367 training images, 100 validation images, and 233 testing images.

### 4.2. Implementation Details

In this paper, all experiments are based on the PyTorch 1.8.0 deep learning framework and conducted on a computer equipped with an NVIDIA GeForce RTX 2080 Ti GPU and CUDA configuration of CUDA 10.2 cuDNN 7.6.5. In the process of model training, the Adam optimizer was used to optimize the parameters of the network. The momentum value was set to 0.9, the weight decay rate was set to 0.0005, and the initial learning rate was set to 0.0006. The initialization strategy of kaiming was used to initialize the network parameters, while the “Poly” learning rate attenuation strategy was adopted. The specific formula was as follows:

(13) $$lr=init\_lr\times {\left(1-\frac{epoch}{max\_epoch}\right)}^{power};$$

here, lr is the new learning rate, init_lr is the benchmark learning rate, the epoch is the number of iterations, and max_epoch is the maximum number of iterations. We have trained 800 epochs in total, and power controls the shape of the curve, which we set to 0.9.

Different batch sizes are designed for two different datasets, Cityscapes and Camvid. Specifically, Cityscapes has a batch size of 8, while Camvid has a batch size of 16. Various data enhancement strategies, including horizontal flipping, clipping, and multi-scale transformation, are used on the training set data. The cross-entropy loss function is used to train the model, and auxiliary losses are added at the end of the third stage of the network to improve the performance, stability, and generalization ability of the model. Therefore, the total loss function can be expressed as:

(14) $$Loss=Loss_{m}+\alpha \times Loss_{a};$$

here, $Loss_{m}$ is the main loss function of the model, the α is the weight coefficient of the auxiliary loss function. The two losses are indicated in Figure 3. In order to obtain the best segmentation model, experiments were conducted to verify the different influences on model performance, as shown in Table 2.

In subsequent experiments, several objective evaluation metrics were used to evaluate the proposed network performance, including mean intersection over union (mIoU), number of model parameters, floating-point operations (GFLOPs), and frames per second (FPS), which is the number of images processed per second. These indicators are used to comprehensively evaluate the performance of the network in different aspects.

### 4.3. Ablation Study

#### 4.3.1. Ablation Experiment of Encoder

**Network depth:** Downsampling is very important in image processing. It can reduce computational complexity, save storage space, suppress noise and enhance receptive field, and improve the efficiency and accuracy of image processing algorithms. However, a large downsampling factor can speed up the processing speed and reduce the resource requirements, but it may lead to a decline of image quality. On the contrary, a smaller downsampling factor can retain more details but may increase the computational complexity. So, it is necessary to balance the image quality and processing speed when selecting the downsampling factor.

In our model, when using the MBRU module to process a quarter of the high-resolution feature map, it only takes up 0.02 M of memory resources, and the amount of calculation reaches 0.65 GFLOP, which requires high computing power and is challenging in obtaining high-level semantic information. At one-sixteenth resolution, the amount of computation is reduced to 0.52 GFLOP. However, the parameters are increased to 0.27 M, which is also not applicable to resource-constrained mobile devices. However, at the one-eighth resolution, we have balanced the computational complexity and the number of parameters. Each MBRU module requires 0.55 GFLOP of computation and 0.07 M of storage resources. It should be noted that these data are only for a single MBRU module, and our network needs to stack multiple MBRU modules, which will make the gap more obvious. Therefore, in order to balance the performance and resource consumption of the model to a certain extent, we choose to process the feature map by downsampling the input to one-eighth of the resolution.

Following this setup, the number of MBRUs in FAHDNet’s second and third stages significantly affects the segmentation performance of the model. An appropriate number of MBRU modules can ensure multi-level features, rich semantic information, and appropriate spatial resolution, which is crucial for the accuracy and efficiency of semantic segmentation tasks. By setting up different numbers of MBRU modules and comparing the mIoU, number of model parameters, and FPS of the model, the optimal number configuration is explored, as shown in Table 3 (Network depth).

As can be seen from the table, adding MBRU modules in the second stage does not significantly improve the performance, but increases the computational burden, resulting in lower segmentation speed. For example, when comparing row 2 and row 5 in the table, when the number is increased to 4, the accuracy is only improved by 0.2%, but the speed is significantly decreased. Therefore, in the second phase, we choose to use two MBRU modules in pursuit of faster speeds. In the third phase, increasing the number of MBRU modules can significantly improve performance. When eight MBRU modules are used, the accuracy can reach 70.6%, while the speed is also fast enough. However, when we increased the number of MBRU modules to 10, too many convolutional modules led to overfitting and increased the computational burden, resulting in a decrease in accuracy and segmentation speed. Hence, we chose to use 8 MBRU modules to balance the need between speed and performance.

**Dilation rates:** In order to gain a deeper understanding of the impact of different dilation rates on the feature extraction capability of the MBRU module at different scales, we conducted experimental verification, as shown in Table 3 (dilation rates). From the data in the table, we can observe four sets of experiments with different dilation rates. In the columns of parameter count (Params (M)) and frames per second (FPS), all variant models had a parameter count of 0.82 M, and FPS ranged from 130 to 142. The differences among the compared models were not significant. Thus, different dilation rate settings have little impact on parameter count and forward propagation speed in our model. However, it can also be observed from the table that the maximum difference in segmentation accuracy among the model sets was 2.1%. Therefore, different dilation rate settings have a significant impact on segmentation accuracy. Hence, selecting an appropriate dilation rate is crucial for accurate segmentation results.

Specifically, the segmentation results of the first group are the worst because of their smaller dilation rates, limiting the receptive field of each pixel, allowing the model to only see information within a relatively small range. In contrast, the receptive fields in the subsequent three groups are larger, and their accuracy is significantly better than that of the first group. It is evident that enlarging the receptive field helps reduce missegmentation in segmentation and improve segmentation accuracy. In the second and third groups, MBRU modules with the same dilation rates were stacked, but the results of the second group are better than those of the third group. This is because the third group continuously uses the same dilation rate four times, limiting the local perception range of the network, resulting in poorer performance in handling certain local features. The second group, on the other hand, addresses this issue by stacking two sequentially increasing dilation convolutions, achieving better results. However, the fourth group, which uses mixed dilation rates, performs slightly worse than the second group. This is because the fourth group introduces additional complexity and makes it difficult to adjust hyperparameters, without improving performance.

Overall, these experimental results emphasize the need to balance different factors when selecting dilation rates to achieve the best segmentation performance.

**Main modules:** To verify the effectiveness of the MBRU module, we replaced it and analyzed the model’s performance. We used the DAB module, SS-nbt module, and EAR module to replace the MBRU module, as shown in Table 3 (main modules). It can be observed that, when we replaced the MBRU module with the DAB module, the accuracy of the DAB variant network was significantly lower than that of the other modules. This suggests that channel interaction contributes to the network’s ability to learn more robust and generalized features, thereby improving segmentation performance. At the same time, the accuracy of the SS-nbt variant network and EAR variant network was also lower than that of FAHDNet, and both of these networks had higher parameter counts and slower speeds compared to ours. This indicates that the MBRU module combines the advantages of the SS-nbt module and the EAR module and optimizes them to achieve a more efficient and effective MBRU module.

**SIC module:** To validate the compensation function of the SIC module for spatial information, we conducted a set of comparative experiments. In the experiments, we removed the SIC module from the encoder and compared the model’s performance in various aspects, as shown in Table 3 (SIC module). We observed that, without the SIC module for information compensation, the model’s segmentation accuracy decreased by 0.8%. Although there was a slight improvement in inference speed, the loss in accuracy was more significant, indicating that the SIC module is necessary for improving model performance.

#### 4.3.2. Ablation Experiment of Decoder

In the decoder, we utilize the GA module to guide feature upsampling, and we conducted a series of experiments to validate its effectiveness, as shown in Table 4. This GA module employs an attention mechanism to give more weight to useful information. In the table, we first assess the network’s performance without the GA module, then evaluate it by introducing the GA module separately in the second and third stages of the decoder and, finally, we use the GA module simultaneously in both stages. The experimental results indicate that introducing the GA module alone improves the model’s performance. However, the most notable observation is that, when using the GA module simultaneously, the network’s accuracy improves by 3.4% compared to when the GA module is not introduced, further confirming the significant enhancement of the GA module on model performance.

### 4.4. Compare with the Existing Model

In this section, the efficacy of the proposed FAHDNet is compared to that of several state-of-the-art models. We report four metrics derived from the Cityscapes and Camvid datasets.

#### 4.4.1. Comparison on Cityscapes

In Table 5, our algorithm is compared with state-of-the-art methods. In terms of similar segmentation accuracy, although our model has a lower accuracy compared to DSANet and DFFNet by 0.4% and 0.8%, respectively, we can observe that DSANet achieves only 34 FPS, and DFFNet achieves only 62.5 FPS, which is approximately a quarter and half of our model’s speed, respectively. Additionally, their parameter counts are several times higher than ours. Therefore, our model is more suitable for scenarios with limited resources and higher real-time performance requirements. Methods with similar model parameters, such as Fast-SCNN, FRNet, and ContexNet, do not perform as well in segmentation as FAHDNet, indicating our algorithm’s advantage under similar resource constraints. While ICNet achieves a similar level of accuracy to our network, its parameter count is 30 times higher than FAHDNet. In comparison, although DFANet’s accuracy is similar to FAHDNet, its parameter count is 10 times higher, demonstrating that FAHDNet utilizes parameters very efficiently. These conclusions indicate that FAHDNet strikes a balance between performance and efficiency, especially achieving higher inference speed with lower resource consumption, making it more competitive in practical applications.

**Figure 9:**
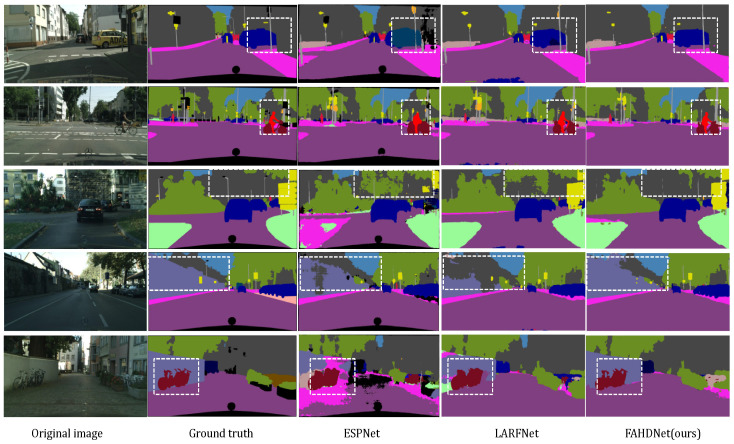
Visual comparisons in terms of the Cityscapes validation set. From left to right are original image, ground truth, segmentation outputs from ESPNet, LARFNet, and our FAHDNet. The white dashed block diagram highlights the important contrasting parts.

The visualization results of FAHDNet on the Cityscapes validation set are shown in Figure 9. From left to right in the figure, you can see the original input image, the annotated image, ESPNet’s result, LARFNet’s result, and FAHDNet’s result. To enable readers to visually identify differences and enhance their understanding and comparison of various features, performances, or results, we use dashed boxes to highlight the vital contrasting sections. From the image, it is evident that, due to ESPNet’s pursuit of lightweight design and its shallow network depth, it falls short in terms of extracting semantic information. This results in category segmentation errors in both the third and fifth rows, as it fails to accurately classify building and wall labels. However, when compared to LARFNet, FAHDNet exhibits a significantly better segmentation performance in terms of details and object boundaries. For example, in the last line, the outline of the bicycle is not segmented, while our network displays it more clearly.

Table 6 displays the segmentation accuracy of the network on the Cityscapes dataset for each class. From the bold font in the table, it is evident that, out of the 21 metrics, we achieved the highest performance in 14, highlighting our network’s overall segmentation superiority. In particular, when compared to other networks, especially the “train” label, our network outperformed them by more than 10%. Among the compared models, a few classes had lower segmentation accuracy than ICNet. ICNet divides the image into different resolution levels, processes them using different network branches, and then combines the information from different resolution levels. This multi-scale processing method enables ICNet to maintain high-resolution details and global semantic information simultaneously, better process multi-scale information, and make ICNet produce better results in “terrain” and “motorcycle” segmentation. However, this multi-branch structure increases the complexity of the network and reduces inference speed, achieving only 30.3 FPS. In contrast, our method utilizes a single branch. It leverages the MBRU module to extract multi-scale information and the SIC module to compensate for spatial information from the original image. Our method simplifies the network structure while achieving a fast inference speed of up to 135 FPS. Additionally, our model has fewer parameters while maintaining the same level of accuracy. Compared with our method, the segmentation speed of ICNet is only about one-quarter of ours, so it is not suitable for scenes requiring a high real-time performance.

#### 4.4.2. Comparison on Camvid

The results on the Camvid dataset are shown in Table 7. FAHDNet achieves a processing speed of 335 FPS for 360×480 resolution Camvid images, with an average intersection over union of 67.2%. The table shows that, although FAHDNet and FRNet have similar parameters and accuracy, FRNet’s inference speed is only one-third of FAHDNet’s. Compared to DFANet, BiSeNet, and CGNet, which use high-resolution inputs of 720×960, our model has inputs with half the resolution. However, the data in the table demonstrate that our model performs significantly better than theirs.

**Figure 10:**
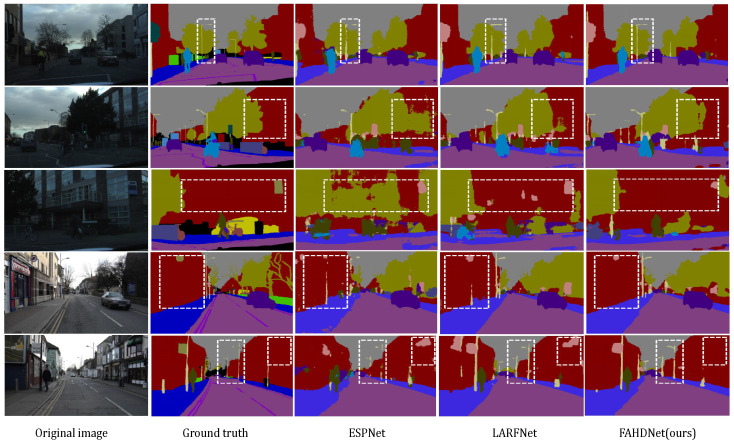
Visual comparisons in terms of the Camvid validation set. From left to right are original image, ground truth, and segmentation outputs of ESPNet, LARFNet, and our FAHDNet. The white dashed block diagram highlights the important contrasting parts.

The visualized results of the Camvid validation set are shown in Figure 10. From left to right are the original image, ground truth, segmentation outputs of ESPNet, LARFNet, and our FAHDNet. Also use the dotted line box to highlight the contrast part, so as to more clearly understand and compare the semantic segmentation results between different models. As can be seen from the first row of the figure, compared with ESPNet, FAHDNet has a superior segmentation effect in terms of details, and its segmentation graph has less noise and has better robustness.

## 5. Conclusions

In this paper, we propose a real-time semantic segmentation network named FAHDNet, which aims to achieve low latency and efficient data processing while maintaining high accuracy. FAHDNet considers the needs of hardware and application scenarios, and employs a variety of technologies to improve the quality of image segmentation while maintaining real-time performance. In the encoder phase, FAHDNet uses the lightweight MBRU module to extract rich semantic information, while the SIC module provides spatial details. These modules help the network to efficiently capture important features of the image. In the decoder stage, FAHDNet adopts the GA-guided hierarchical decoding structure, which helps to fuse semantic information and spatial information, thus improving the wrong segmentation of small targets and obtaining an image segmentation model with a superior performance. In summary, FAHDNet is a deep learning model that combines attention mechanisms, multi-scale information integration, and step-by-step decoding mechanisms to improve the accuracy of image segmentation, while being suitable for application scenarios requiring low latency and efficient processing. 

## Figures and Tables

**Table 1:** FAHDNet structure details. The input size is 512 × 1024, and ‘C’ refers to the number of output categories.

Type	Stage	Layer	Operator	Output Size
Encoder	Stage 1	1	Conv unit	256 × 512 × 32
		2	SIC	256 × 512 × 32
		3	SCA	256 × 512 × 32
	Stage 2	4	Conv unit	128 × 256 × 64
		5	SIC	128 × 256 × 64
		6–7	MBRU	128 × 256 × 64
		8	SCA	128 × 256 × 64
	Stage 3	9	Conv unit	64 × 128 × 128
		10	SIC	64 × 128 × 128
		11–18	MBRU	64 × 128 × 128
		19	SCA	64 × 128 × 128
Decoder		20	GA	64 × 128 × 128
		21	Upsampleing	128 × 256 × 128
		22	Conv unit	128 × 256 × 64
		23	GA	128 × 256 × 64
		24	Upsampleing	256 × 512 × 64
		25	Seghead	512 × 1024 × C

**Table 2:** Segmentation accuracy corresponding to different weight coefficients (α). Black bold indicates the parameter settings used in our proposed method.

α	0	0.25	**0.5**	0.75	1
mIoU(%)	69.7	70.1	**70.6**	70.2	69.5

**Table 3:** Results of encoder-related ablation studies. The bold part in the table represents our network settings. ‘w/o’ represents not containing this module, ‘w’ represents containing this module. Black bold indicates the parameter settings used in our proposed method.

Type	Setting	FPS	Params (M)	mIoU(%)
Network depth	(2,6)	152	0.72	69.2
(Stage 2, Stage 3)	**(2,8)**	**135**	**0.82**	**70.6**
	(2,10)	123	0.87	70.4
	(4,6)	131	0.75	69.5
	(4,8)	119	0.84	70.6
	(4,10)	108	0.89	70.1
Dilation rates	(2,5;2,5,7,9,2,5,7,9)	142	0.82	68.5
	**(2,5;2,4,8,8,16,16,32,32)**	**135**	**0.82**	**70.6**
	(2,5;4,4,4,4,16,16,16,16)	138	0.82	69.7
	(2,5;2,4,8,16,4,8,16,32)	138	0.82	70.2
main modules	DAB module	161	0.58	67.8
	SS-nbt module	123	0.92	70.2
	EAR module	102	0.89	70.8
	**MBRU module**	**135**	**0.82**	**70.6**
SIC module	SIC-w/o	159	0.80	69.6
	**SIC-w**	**135**	**0.82**	**70.6**

**Table 4:** Results of decoder-related ablation studies. The bold part in the table represents our network settings.

Encoder	Decoder		FPS	Params (M)	mIoU(%)
	Stage 2	Stage 3			
✓	-	-	167	0.70	67.9
✓	✓	-	156	0.72	68.8
✓	-	✓	151	0.80	70.0
✓	✓	✓	135	0.82	70.6

**Table 5:** Comparison of experimental results of FAHDNet with other advanced models on the Cityscapes test set. ‘-’ indicates that no relevant data are given in the original paper.

Method	Input Size	Pretrain	Params (M)	GFLOPs	FPS	mIoU(%)
SegNet [16]	360×480	ImageNet	29.5	286	15	56.1
ENet [19]	360×480	No	0.36	3.8	135.4	58.3
ESPNet [29]	512×1024	No	0.36	-	113	60.3
CGNet [12]	1024×2048	No	0.49	-	17.6	64.8
Fast-SCNN [11]	1024×2048	No	1.11	-	123.5	68.0
BiSeNet [9]	768×1536	ImageNet	5.80	14.8	106	68.4
LARFNet [42]	512×1024	No	0.72	-	127	69.2
ICNet [13]	1024×2048	ImageNet	26.5	28.3	30.3	69.5
ERFNet [17]	512×1024	ImageNet	2.06	-	41.7	69.7
AGLNet [43]	512×1024	No	1.12	13.88	52	70.1
DABNet [14]	512×1024	No	0.76	-	104.2	70.1
DFANet [38]	512×1024	No	7.8	1.7	160	70.3
FRNet [44]	512×1024	No	1.01	12.9	125	70.4
CIFRENet [45]	512×1024	No	1.9	16.5	34.5	70.9
DFFNet [46]	512×1024	No	1.9	6.9	62.5	71.0
DSANet [47]	512×1024	No	3.47	37.4	34	71.4
JPANet [27]	512×1024	ImageNet	3.49	10.9	110	71.6
Ours	512 × 1024	No	0.82	9.98	135	70.6

**Table 6:** Class mIoU scores on Cityscapes test set for the per-class category. Bold displays the highest segmentation accuracy in the current class.

Method	Road	Sidewalk	Building	Wall	Fence	Pole	Traffic Light	Traffic Sign	Vegetation	Terrain	Sky	Person	Rider	Car	Truck	Bus	Train	Motorcycle	Bicycle	Class IoU	Category IoU
ENet [19]	96.3	74.2	75.0	32.2	33.2	43.4	34.1	44.0	88.6	61.4	90.6	65.5	38.4	90.6	36.9	50.5	48.1	38.8	55.4	58.3	80.4
ESPNet [29]	97.0	77.5	76.2	35.0	36.1	45.0	35.6	46.3	90.8	63.2	92.6	67.0	40.9	92.3	38.1	52.5	50.1	41.8	57.2	60.3	82.2
CGNet [12]	95.5	78.7	88.1	40.0	43.0	54.1	59.8	63.9	89.6	67.6	92.9	74.9	54.9	90.2	44.1	59.5	25.2	47.3	60.2	64.8	85.7
ERFNet [17]	97.7	81.0	89.8	42.5	48.0	56.3	59.8	65.3	91.4	68.2	94.2	76.8	57.1	92.8	50.8	60.1	51.8	47.3	61.7	68.0	86.5
ICNet [13]	97.1	79.2	**98.7**	43.2	**48.9**	**61.5**	**60.4**	63.4	91.5	**68.3**	93.5	74.6	56.1	92.6	51.3	72.7	51.3	**53.6**	**70.5**	69.5	86.4
Ours	**98.0**	**81.7**	90.7	**47.2**	46.3	54.8	59.6	**65.9**	**91.6**	67.2	**94.7**	**78.3**	**58.8**	**93.6**	**56.8**	**75.5**	**63.3**	52.8	63.8	**70.6**	**86.8**

**Table 7:** Comparison of experimental results of FAHDNet with other advanced models on the Camvid test set. ‘-’ indicates that no relevant data are given in the original paper.

Method	Input Size	Params (M)	FPS	mIoU(%)
ENet [19]	360×480	0.36	227	51.3
SegNet [16]	360×480	29.5	46	55.6
ESPNet [29]	360×480	0.68	205	62.6
ERFNet [17]	360×480	2.06	164	63.7
CIFRENet [45]	360×480	1.9	34.5	64.5
DFANet [38]	720×960	7.8	120	64.7
DFFNet [46]	360×480	1.9	62.5	64.7
BiSeNet [9]	720×960	5.8	-	65.6
CGNet [12]	720×960	0.5	-	65.6
JPANet [30]	360×480	3.49	110	67.0
ICNet [13]	360×480	26.5	27.8	67.1
FRNet [44]	720×960	1.01	125	67.4
Ours	360×480	0.82	335	67.2

## Data Availability

Data will be made available on request.
